# Antitumour and antiangiogenic effects of IDN 5390, a novel C-seco taxane, in a paclitaxel-resistant human ovarian tumour xenograft

**DOI:** 10.1038/sj.bjc.6601730

**Published:** 2004-03-09

**Authors:** G Petrangolini, G Cassinelli, G Pratesi, M Tortoreto, E Favini, R Supino, C Lanzi, S Belluco, F Zunino

**Affiliations:** 1 Department of Experimental Oncology, Istituto Nazionale Tumori, via Venezian 1, 20133 Milan, Italy; 2 Department of Veterinary Pathology, Hygiene and Public Health, University of Milan, 20133 Milan, Italy

**Keywords:** taxanes, IDN 5390, angiogenesis, MDR, *in vivo* systems

## Abstract

IDN 5390 is a novel C-seco taxane analogue selected for preclinical development on the basis of its antimotility activity on endothelial cells, antitumour efficacy in a large panel of human tumour xenografts and high tolerability in mouse. On the basis of oral availability, IDN 5390 is suitable for protracted administration schedules. Such a treatment schedule has been reported as the most appropriate to exploit the antiangiogenic effects of cytotoxic drugs. An ability to downregulate angiogenesis-related growth factors in tumour cells has been described for IDN 5390. The aim of the study was to investigate the antitumour and antiangiogenic potential of oral IDN 5390 on a human ovarian carcinoma xenograft, the INT.ACP/PTX, resistant to paclitaxel (PTX). Such tumour line was derived *in vivo* from a cisplatin-resistant tumour line, the A2780/DDP, which is sensitive to PTX. Compared to the parental cells, INT.ACP/PTX cells exhibited a high level of Pgp expression, resulting in a reduced *in vitro* sensitivity to both PTX and IDN 5390. The INT.ACP/PTX tumour xenograft was still resistant to PTX, but responsive to IDN 5390, when delivered per os, by a daily prolonged schedule. A direct effect on tumour cells, allowed by the high tolerability of the compound in mouse, cannot be excluded *in vivo*. Immunohistochemical analysis indicated a significant reduction of microvessel density in IDN 5390-treated tumours, lasting till 7 days after the last drug administration. Thus, a prolonged inhibitory effect on tumour angiogenesis is consistent with the persistent growth control of INT.ACP/PTX tumour achieved by IDN 5390. On the contrary, the low tolerability and the limited oral availability of conventional taxanes do not allow an easy feasibility of such treatment regimen. Thus, the tolerability profile of IDN 5390 in preclinical systems and its efficacy in PTX-resistant tumours support the therapeutic interest for its clinical development, with particular attention to oral daily prolonged schedules.

Taxanes are effective antitumour agents characterised by a unique mechanism of action, that is, microtubule polymerisation resulting in cell death following arrest of cell cycle in M phase and formation of aberrant mitoses (Horwitz *et al*, 1993; Jordan *et al*, 1993). In addition to the cytotoxic effects on tumour cells, effects on angiogenesis have been reported for paclitaxel (PTX) and docetaxel, the two taxanes currently used in clinics (Verweij *et al*, 1994; Rowinsky, 1997). These effects have been related to the ability of inhibiting endothelial cell motility (Belotti *et al*, 1996; Sweeney *et al*, 2001) and downregulating angiogenesis-related growth factors (Cassinelli *et al*, 2002). The novel C-seco taxane IDN 5390, which is currently under study in preclinical systems, was originally selected for its potent antimotility activity and low cytotoxicity in endothelial cells (Taraboletti *et al*, 2002). *In vivo* studies in human tumour xenografts have indicated a unique profile of tolerability and antitumour activity for IDN 5390 when administered by a daily schedule for a prolonged time (Pratesi *et al*, 2003).

In recent years, many preclinical studies have indicated the possibility of using cytotoxic drugs as antiangiogenic agents for the antitumour therapy. Chronic and frequent treatment schedules have been reported as the most appropriate to exploit the antiangiogenic effect of cytotoxic drugs, compared to the high-dose/intermittent-schedule mainly targeting tumour cells (Hanahan *et al*, 2000; Miller *et al*, 2001). Conventional antitumour drugs delivered according to the so-called ‘metronomic chemotherapy’ were effective even against drug-resistant tumours (Browder *et al*, 2000; Fidler and Ellis, 2000). When administered according to a frequent and prolonged schedule, IDN 5390 is active against all human tumour xenografts investigated, including the PTX-resistant ones (Pratesi *et al*, 2003).

The aim of the study was to further investigate the mechanisms responsible for the antitumour activity of IDN 5390 in PTX-resistant human tumour xenografts and, in particular, in the ovarian carcinoma INT.ACP/PTX. Such tumour line was derived *in vivo* by one single s.c. growing A2780/DDP tumour xenograft (cisplatin-resistant) after treatment with PTX. The mechanisms of resistance of these tumour cells and the *in vivo* antiangiogenic effect of the drug were investigated. The results are consistent with a contribution of antiangiogenic effects in the antitumour efficacy of the taxane analogue.

## MATERIALS AND METHODS

### Drugs, tumour and cells

**Figure 1:**
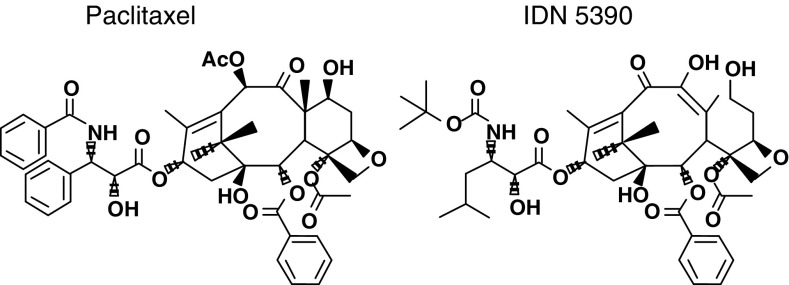
Chemical structures of paclitaxel and IDN 5390.

IDN 5390, (13-(*N*-Boc-*β*-isobutylisoserinyl)-10*β*-dehydro-C-seco-10-deacetylbaccatin III) and PTX, used as reference drug, were provided by Indena S. p.a. (Milan, Italy) (Figure 1

). For *in vitro* studies, drugs were dissolved in dimethylsulphoxide (DMSO) at 2 mg ml^−1^ and diluted in culture medium (DMSO final concentration 0.25%). For *in vivo* studies, PTX was dissolved by adding absolute ethanol and Cremophor ELP (both 5% of the final volume) (Polizzi *et al*, 1999), whereas IDN 5390, dissolved in Polysorbate 80, was diluted just before use by adding 0.9% NaCl to the final concentration of 10% v v^−1^ (Pratesi *et al*, 2003).

The study was performed on the A2780 human ovarian carcinoma cell line and two variants, that is, the A2780/DDP cell subline, selected *in vitro* for resistance to cisplatin (Beherens *et al*, 1987), and the INT.ACP/PTX cell subline. The latter variant was derived from a single A2780/DDP tumour xenograft which, unexpectedly, was not responding to PTX treatment *in vivo*. Indeed, the A2780/DDP tumour line is highly sensitive to PTX treatment (Polizzi *et al*, 1999). The line was maintained either by s.c. passages of tumour fragments (tumour line) or as cell culture (cell line). A2780/DX, selected *in vitro* for acquired resistance to doxorubicin, was used as positive control for the Pgp expression analysis.

All cell lines were maintained in RPMI 1640 (Bio-Whittaker Verviers, Belgium) supplemented with 10% foetal calf serum (Life Technologies, Gaithersburg, MD, USA) in 5% CO_2_ atmosphere.

### *In vitro* studies

Tumour cell sensitivity to IDN 5390 and PTX was evaluated by cell growth inhibition assay. Cells were seeded in six-well plates in duplicate and after 24 h exposed to the solvent or to the drugs at different concentrations. After 72 h, cells were trypsinised and counted by a Coulter Counter (Coulter Electronics, Luton, UK). The concentration able to inhibit cell proliferation by 50% (IC_50_) was derived by dose/effect plots. Resistant Index (RI) was assessed as the ratio between the drug IC_50_ in each cell subline and in the parental A2780 cell line.

For the MDR phenotype characterisation, the Pgp expression was determined by immunofluorescence (Gottesman and Pastan, 1993). Cells (10^6^) were trypsinised and washed in PBA (phosphate-buffered saline (PBS) and 1% bovine serum albumin (BSA)). Cells were then incubated in 100 *μ*l of PBA containing 10 *μ*l of gp170-FITC antibody (YLEM, Rome, Italy). After washing in PBS, expression of the protein was assessed as fluorescence intensity using a FACScan (Becton Dickinson, Mountain View, CA, USA).

Bcl-2 expression was examined in whole-cell extracts prepared as previously reported in Pratesi *et al* (2003). Briefly, equal amounts of proteins were separated by SDS–PAGE and transferred onto nitrocellulose sheets. Then filters were incubated with mouse monoclonal anti-Bcl-2 antibody (Santa Cruz Biotechnology, CA, USA) or with rabbit polyclonal antiactin (Sigma, St Louis, MO, USA). Immunocomplexes were visualised by the Pierce Super Signal System (Pierce, Rockford, IL, USA).

### *In vivo* studies

All the experiments were carried out using adult (8–10 weeks old) female athymic CD-1 nude mice (Charles River, Calco, Italy). Mice were maintained in laminar flow rooms at constant temperature and humidity, with free access to sterilised food and water. Experimental protocols were approved by the Ethic Committee for Animal Experimentation of our Institute (Istituto Nazionale per lo Studio e la Cura dei Tumori, Milan, Italy), according to the United Kingdom Coordinating Committee on Cancer Research Guidelines (Workman *et al*, 1998).

The INT.ACP/PTX tumour was maintained in line by subsequent fragment implants, as previously described (Pratesi *et al*, 1989). At each passage mice were treated with PTX (i.v., 36 mg kg^−1^, 7 days after tumour inoculum), and in such conditions tumour weight inhibition (TWI) induced by the optimal PTX regimen was lower than 50 *vs* a 94% inhibition in the A2780/DDP tumour line (Polizzi *et al*, 1999).

Drug solutions of 3.6 and 9 mg ml^−1^ were prepared, as described above, for PTX and IDN 5390, respectively, corresponding to 36 and 90 mg kg^−1^ for an administration volume of 10 ml kg^−1^ of body weight. Larger volumes were injected for higher doses.

For experimental purposes, tumour fragments were s.c. implanted in the right flank of mice. Tumour growth was followed by biweekly measurements of tumour diameters with a Vernier caliper. Tumour weight (TW) was calculated according to the formula: TW (mg)=tumour volume (mm^3^)=*d*^2^ × *D*/2, where *d* and *D* are the shortest and the longest diameter, respectively. Mice bearing tumours of 250–300 mg were treated with IDN 5390 and PTX, according to different treatment routes (i.v., s.c. or p.o.) and schedules (daily, i.e. qd × 8, or intermittent, i.e. q4d × 3). The first and the last day of treatment were the same for the two schedules. At 1 and 7 days after treatment end, three mice/group were killed by cervical dislocation. Tumours were excised, weighed, fixed in zinc fixative and stained with standard haematoxylin—eosin in order to study the overall tissue morphology. Angiogenesis was assessed by immunohistochemistry (IHC), staining blood vessels with a rat anti-mouse CD31/PECAM-1 monoclonal antibody (kindly supplied by Dr A Vecchi, Mario Negri Institute, Milan, Italy), as previously described (Cassinelli *et al*, 2002). Microvessel density (MVD) was determined using a WebSlide Browser software. Briefly, microvessels were quantified within six random fields (0.159 mm^2^ fields, × 200 magnification) selected for high vascularisation (hot spot areas). Microvessel density was expressed as mean number±s.d. Percentage MV inhibition (MVI%) in drug-treated *vs* control mice was calculated as MVI%=100–(mean MVD in treated/mean MVD in control tumours × 100). Neither vessel lumen nor red blood cells were used to define a microvessel. Scoring of histological tumour sections was performed by two independent observers, without knowledge of the experimental group, with an interobserver reproducibility >95%.

Drug efficacy was assessed, at the last day of treatment, as percentage TW inhibition (TWI%) expressed as TWI%=100–(mean TW treated/mean TW control × 100).

For statistical analysis, TW and MV number were compared in treated *vs* control mice by the unpaired Student's *t*-test (two-tailed).

## RESULTS

### Chemosensitivity and drug resistance

**Figure 2:**
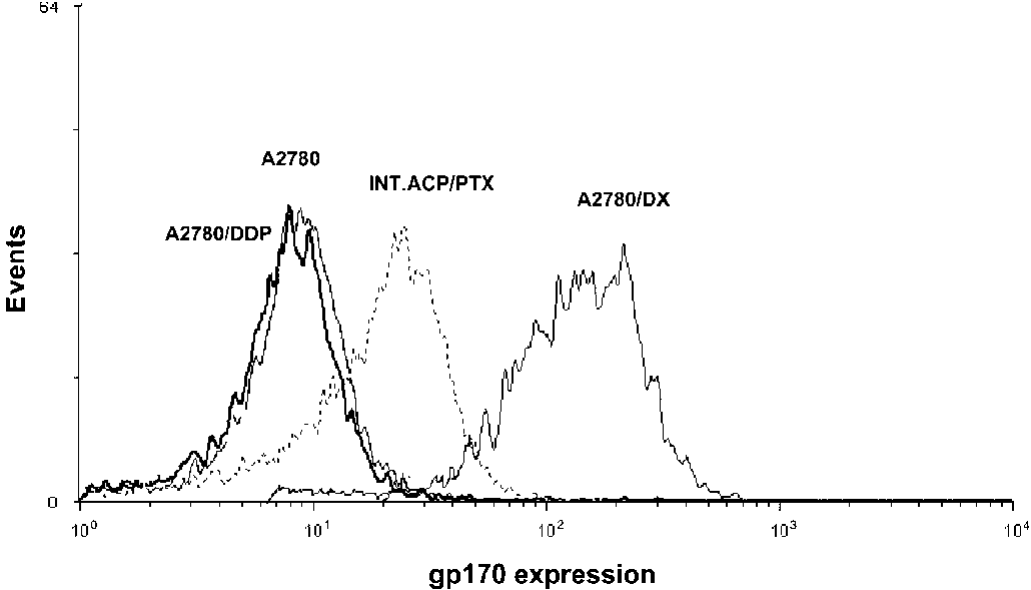
Pgp expression in the A2780 and its variant cell lines. A2780/DX cells were used as positive control. Protein expression was assessed as fluorescence intensity in logarithmically growing cells stained with gp170-FITC antibody. One experiment representative of two for each cell line is reported.

**Figure 3:**
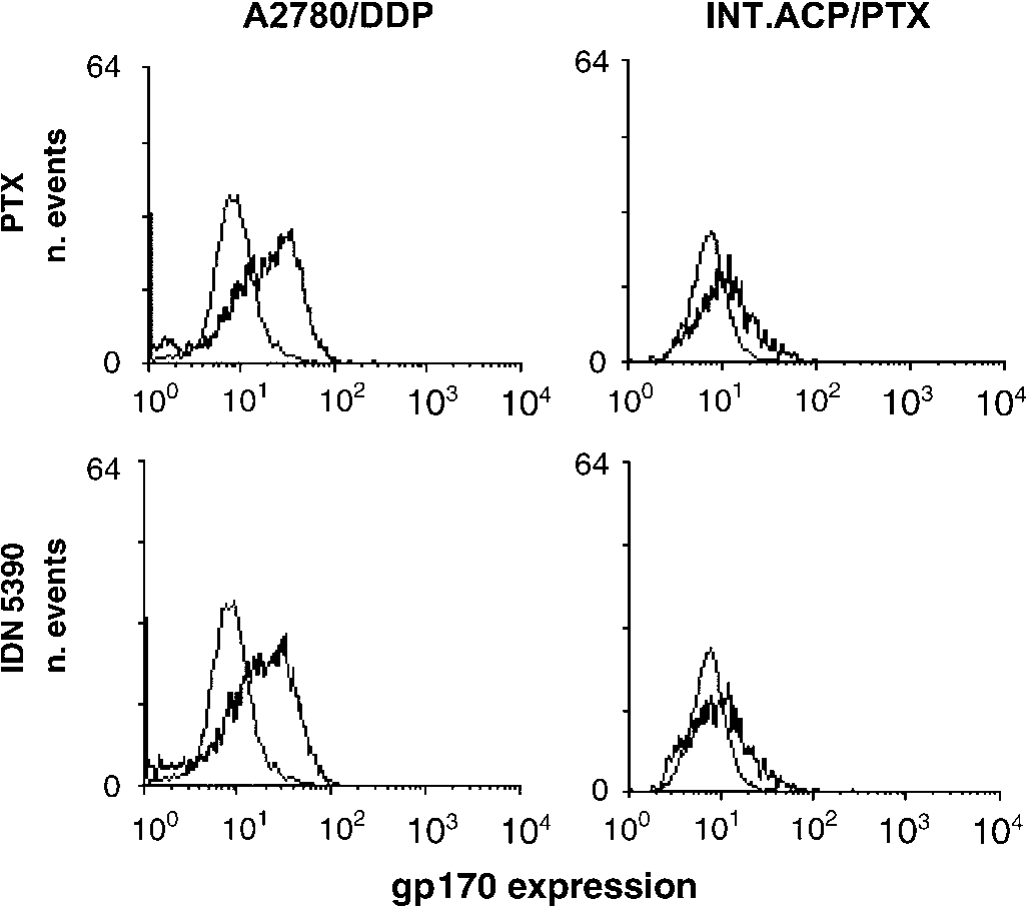
Pgp expression in A2780/DDP and INT.ACP/PTX cell lines treated with PTX or IDN 5390. Protein expression was assessed as fluorescence intensity in cells treated with the IC_80_ of each drug for 72 h and stained with gp170-FITC antibody. One experiment representative of two for each cell line is reported.

Pgp overexpression is a frequent mechanism of the cellular resistance to hydrophobic drugs, including PTX (Horwitz *et al*, 1993; Zunino *et al*, 1999). Thus, the Pgp expression levels were investigated by FACS analysis in the parental A2780 and in its variant cell lines (Figure 2

). Pgp was expressed at low level in A2780 and A2780/DDP cells (mean fluorescence intensity being 8.5 and 7.9, respectively), whereas an increased expression was found in INT.ACP/PTX cells (mean fluorescence intensity, 25). A2780/DX cells were used as a positive control because of their MDR phenotype (mean fluorescence intensity, 140). Since Pgp expression can be modulated by treatment with drugs that are substrates for the transport system (Gottesman and Pastan, 1993), the protein level was examined after 72 h-treatment with PTX or IDN 5390. As shown in Figure 3

an increased Pgp level was found in A2780/DDP cells after exposure to taxanes, whereas no significant modulation was observed in A2780 (not shown) and INT.ACP/PTX cells.

The antiproliferative effects of PTX and IDN 5390 after 72 h of treatment indicated an inverse correlation between Pgp expression and cell sensitivity to the taxanes (Table 1

**Table 1:** Antiproliferative activity of IDN 5390 and PTX on different human ovarian tumour cell lines

	**PTX**		**IDN 5390**	
	**IC_50_^a^ (nM)**	**RI^b^**	**IC_50_^a^ (nM)**	**RI^b^**
A2780	9.5±1		29±0.5	
A2780/DDP	13.8±1.7	1.5	40.4±7	1.4
INT.ACP/PTX	140±23	15	203±35.5	7
A2780/DX	366±30	38.6	424±44.4	14.5

a IC_50_: drug concentration required to inhibit 50% of cell growth compared to untreated cells (72 h of drug exposure). Mean±s.d. of at least three independent experiments are reported.

b Resistance Index (RI), calculated as the ratio between the IC_50_ of the cell sublines and the IC_50_ of the parental A2780 cell line.

). Thus, A2780/DX and INT.ACP/PTX cells exhibited a reduced sensitivity to both taxanes. However, a lower Resistance Index (RI) was observed for IDN 5390 than for PTX. Co-treatment of INT.ACP/PTX cells with each taxane and the pump-antagonist verapamil (10 *μ*M) resulted in a complete reversal of resistance, with IC_50_ values similar to those in the A2780/DDP cell line, that is, 17.5±1.2 and 57±4 nM for PTX and IDN 5390, respectively (not shown).

**Figure 4:**
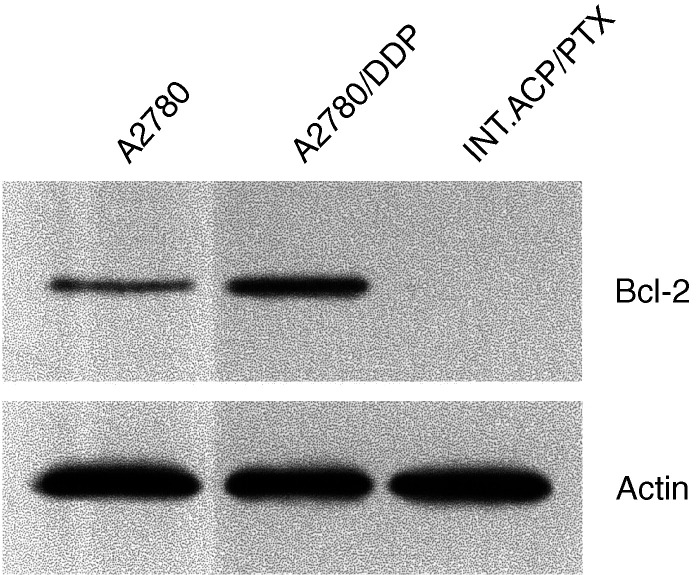
Expression of Bcl-2 protein in A2780 and its variant cell lines. Equal amounts of proteins were separated by SDS–PAGE and analysed by immunoblotting. Control for protein loading by actin is shown.

A differential expression of Bcl-2 protein was detected by Western Blot analysis in the cell lines. A2780/DDP cell lines showed increased basal levels of this protein compared to the parental A2780 cell line. No Bcl-2 protein was detectable in INT.ACP/PTX cells by this technique (Figure 4

).

### *In vivo* studies

We have already reported that the INT. ACP/PTX tumour xenograft is resistant to PTX (less than 50% TWI), but highly sensitive (87% TWI) to IDN 5390, 120 mg kg^−1^, delivered twice a day for 15 days (Pratesi *et al*, 2003). In the present study, the same dose of IDN 5390 was delivered only once a day for eight treatments, and a lower antitumour effect (60% TWI) was achieved (Table 2

**Table 2:** Antitumour and antiangiogenic effects of IDN 5390 and PTX delivered by different treatment schedules against the INT.ACP/PTX human ovarian tumour xenograft

		**Dose (mg kg^−1^)**		**Treatment**			**MVI%^b^**	
**Drug**	**Route**	**Single**	**Total**	**Schedule**	**Days**	**TWI%^a^**	**+1**	**+7**
PTX	s.c.	10	80	Daily × 8	4–11	0††	50**	21
	i.v.	54	162	q3-4d × 3	4,7,11	31†	26*	14
								
IDN 5390	p.o.	120	960	Daily × 8	4–11	60	55**	26*

a Tumour weight inhibition percentage, evaluated 1 day after the last treatment. TW in controls was mg 425±235 (mean±s.d.).

b Tumour Microvessel Inhibition percentage, assessed 1 and 7 days after the last treatment. Microvessel numbers in control tumors were 18±5 and 12±2.6 (mean±s.d.) at 1 and 7 days, respectively.

† *P*<0.05 and

†† *P*<0.005 *vs* IDN 5390-treated mice;

* *P*<0.05 and

** *P*<0.0001 *vs* control tumours, by Student's *t*-test.

). Anyway, IDN 5390 resulted much more effective than PTX delivered in similar conditions (*P*<0.005), or at its best regimen (*P*<0.05).

**Figure 5:**
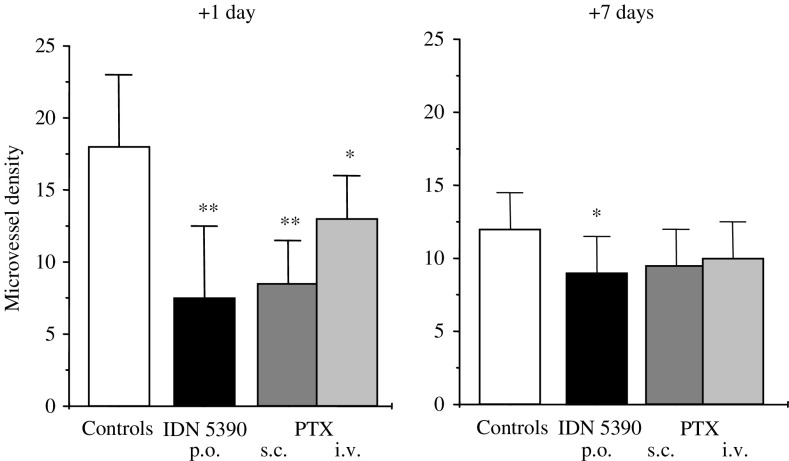
Microvessel density in INT.ACP/PTX tumour xenograft after IDN 5390 or PTX treatment. Tumours were implanted s.c. in mice and treated with: solvent (empty column); IDN 5390 120 mg kg^−1^, per os, daily × 8 (black column); PTX 10 mg kg^−1^, s.c., daily × 8 (dark grey column); PTX 54 mg kg^−1^, i.v., q4d × 3 (light grey column). At 1 or 7 days after last treatment, tumours were surgically removed, and MVD was evaluated by immunoistochemical analysis (see Materials and Methods). Columns represent the mean MVD±s.d. ^*^P<0.05, ^**^P<0.0001 *vs* control tumours, by Students' *t*-test.

A prolonged treatment schedule, which resulted to be the optimal one for the antitumour efficacy of IDN 5390, has been reported as the most suitable for exploiting the antiangiogenic effect of cytotoxic drugs. Thus, the effect on angiogenesis of the novel taxane was assessed in INT.ACP/PTX tumour xenografts (Table 2 and Figure 5

). The MVD analysis performed one day after the last drug treatment (+1) clearly showed that both drugs, delivered by the daily schedule, significantly decreased MVD *vs* control tumours (55 and 50% MVI, respectively; *P*<0.0001 for both). Paclitaxel was administered daily by the s.c. route, due to its very poor oral bioavailability (Sparreboom *et al*, 1997; Polizzi *et al*, 2000). After 1 week (+7), a significant inhibitory effects on MVD was still present only in the IDN 5390-treated tumours (*P*<0.05). Paclitaxel administered i.v. at its best regimen for antitumour activity (high-dose/intermittent schedule) had limited effect on tumour angiogenesis.

## DISCUSSION

Resistance to taxanes involves several mechanisms including mdr gene amplification or overexpression and alterations in drug–target interactions (Horwitz *et al*, 1993). Indeed, mdr-1 gene expression has been associated with the development of PTX resistance in patients with ovarian cancer (Kamazawa *et al*, 2002). In contrast, *β*-tubulin mutations have been reported to be rare in newly diagnosed and in recurrent PTX-resistant human ovarian cancer patients (Lamendola *et al*, 2003). The tumour line investigated in the study, INT-ACP/PTX, was originated following *in vivo* treatment with PTX. Such a model, as far as we know, is the first experimental system where tumour resistance to PTX was acquired *in vivo*, using therapeutic doses of the drug. Our results indicate that in the INT.ACP/PTX tumour cells the resistance to PTX was mediated by expression of Pgp, because tumour cells displayed an increased expression of Pgp protein compared to the parental cell line A2780/DDP. The high susceptibility of A2780/DDP cells to acquire an MDR phenotype was supported by the increased expression of Pgp observed after treatment with taxanes in such cells.

Recently, Bcl-2 downregulation has been reported as another resistance mechanism to PTX (Ferlini *et al*, 2003). The Bcl-2 protein is downregulated in the resistant INT.ACP/PTX cells compared to the A2780/DDP cells. However, the relevance of this finding in the resistant phenotype remains to be defined. Indeed, the almost complete reversal of resistance to PTX by verapamil supports a typical MDR-mediated resistance.

In the present study IDN 5390, 120 mg kg^−1^, administered once a day for 8 days induced a marginal tumour response (60% TWI), whereas a superior antitumour effect (87% TWI) was achieved by the same dose twice a day for 2 weeks (Pratesi *et al*, 2003). Indeed, the very low toxicity of the compound allows very frequent drug administration and very prolonged period of treatment. Indeed, total doses up to 4.5 g kg^−1^, that is, 20-fold higher than those of PTX, were well tolerated (Pratesi *et al*, 2003). Thus, in spite of the recognition by transport systems detected in cell systems (Distefano *et al*, 1998; our results), a contribution of cytotoxic effect in antitumour activity of IDN 5390 could be expected. Indeed, the previously reported plasma levels of IDN 5390 in mouse (*C*_max_, 25 *μ*g ml^−1^) after oral administration of a single dose (120 mg kg^−1^) are high enough to support our interpretation (Pratesi *et al*, 2003).

Tumour growth inhibition by IDN 5390 was associated with a strong reduction of tumour MVD, which was more evident 1 day after treatment end (55% MVI) and still present 1 week after (26% MVI). Such finding was in keeping with other preclinical *in vivo* studies on inhibition of tumour angiogenesis by various cytotoxic drugs delivered by the so-called ‘metronomic chemotherapy’, that is, frequent and prolonged administration schedule in contrast to the conventional high-dose/intermittent-schedule regimen (Hanahan *et al*, 2000; Schirner, 2000; Miller *et al*, 2001). Indeed, the conventional regimen of PTX (i.v., high-dose/intermittent-treatment) compared to the daily schedule, achieved a superior level of antitumour activity (31 *vs* 0% TWI), but a lower effect on MVD. Inhibition of angiogenesis in the INT.ACP/PTX tumour xenograft was achieved also by PTX delivered by the daily s.c. treatment. The reduction on MVD was similar to that achieved by the analogue, but PTX-induced effect was less persistent. No antitumour efficacy was achieved by PTX delivered by these conditions. In spite of a similar ability to inhibit tumour angiogenesis, the limited exposure of the tumour cells to PTX, due to the low drug tolerability *in vivo*, may account for the lack of efficacy of PTX. In addition, the higher level of resistance (RI 15 *vs* 7 for PTX and IDN 5390, respectively) may be a further drawback of PTX. Moreover, considering the very poor oral bioavailability of PTX (Sparreboom *et al*, 1997; Polizzi *et al*, 2000), the s.c. route was used for the daily treatment, and only 10 mg kg^−1^ were tolerated, due to the high vesicant activity of the drug (Pratesi *et al*, 2003). In contrast, the possibility of delivering repeated administrations of IDN 5390 allows keeping tumour angiogenesis under control for long time. The effects of IDN 5390 on tumour angiogenesis *in vivo* are consistent with its ability to inhibit endothelial cell motility (Taraboletti *et al*, 2002) as well as to downregulate the expression of angiogenic growth factors (Pratesi *et al*, 2003). Downregulation of VEGF and bFGF expression, already reported in a glioma cell line (Pratesi *et al*, 2003), was observed even in INT-ACP/PTX cells (not shown). Thus, the responsiveness of the tumour to IDN 5390 may be the result of the contribution of both antiangiogenic and cytotoxic effects.

In conclusion, the relevant antitumour activity of the novel taxane IDN 5390 against a PTX-resistant tumour xenograft is likely to be the result of multiple actions, favoured by the peculiar pharmacological properties of the compound. Indeed, the good tolerability *in vivo* and the oral bioavailability allow exploiting the therapeutic potential of high doses of the drug (possibly allowing direct cytotoxicity) throughout a continuous and prolonged period of treatment (favouring effect on angiogenesis inhibition). Although the therapeutic advantages of IDN 5390 over PTX could be achieved only at relatively high dose levels, the favourable pharmacological profile makes IDN 5390 a promising candidate for clinical development.
